# Influenza A(H1N1)pdm09 Virus with Reduced Susceptibility to Baloxavir, Japan, 2024

**DOI:** 10.3201/eid3105.241123

**Published:** 2025-05

**Authors:** Emi Takashita, Hiroko Morita, Shiho Nagata, Seiichiro Fujisaki, Hideka Miura, Tatsuya Ikeda, Kenichi Komabayashi, Mika Sasaki, Yohei Matoba, Tomoko Takahashi, Naomi Ogawa, Katsumi Mizuta, Sueshi Ito, Noriko Kishida, Kazuya Nakamura, Masayuki Shirakura, Shinji Watanabe, Hideki Hasegawa

**Affiliations:** Author affiliations: National Institute of Infectious Diseases, Tokyo, Japan (E. Takashita, H. Morita, S. Nagata, S. Fujisaki, H. Miura, N. Kishida, K. Nakamura, M. Shirakura, S. Watanabe, H. Hasegawa); Yamagata Prefectural Institute of Public Health, Yamagata, Japan (T. Ikeda, K. Komabayashi, M. Sasaki, Y. Matoba, T. Takahashi, N. Ogawa, K. Mizuta); Ito Clinic, Yamagata (S. Ito)

**Keywords:** influenza virus, influenza, viruses, cap-dependent endonuclease inhibitor, baloxavir marboxil, baloxavir acid, drug resistance, antimicrobial resistance, respiratory infections, A(H1N1)pdm09, I38N substitution, polymerase acidic protein, Japan

## Abstract

Influenza A(H1N1)pdm09 virus carrying an I38N substitution was detected in an untreated teenager in Japan. The I38N mutant virus exhibited reduced susceptibility to baloxavir but remained susceptible to neuraminidase inhibitors and showed reduced growth capability. Monitoring antiviral drug susceptibility of influenza viruses is necessary to aid public health planning and clinical recommendations.

The cap-dependent endonuclease inhibitor baloxavir marboxil is approved in Japan for the treatment and prophylaxis of influenza virus infection in young patients. For patients >12 years of age, the approved doses are 80 mg for those weighing >80 kg and 40 mg for those weighing <80 kg. For patients <12 years of age, the approved doses are 40 mg for those weighing >40 kg, 20 mg for those weighing 20 to <40 kg, and 10 mg for those weighing 10 to <20 kg. The Japan Pediatric Society did not recommend use of baloxavir in children <5 years of age during the 2023–24 influenza season (https://www.jpeds.or.jp/uploads/files/20231122_influenza.pdf). Baloxavir acid, an active form of baloxavir marboxil, binds to the polymerase acidic (PA) protein endonuclease domain and inhibits RNA cleavage by the PA cap-dependent endonuclease (*1*). Amino acid substitutions at position 38 in the PA protein are recognized as treatment-emergent mutations associated with reduced susceptibility to baloxavir (*2*,*3*) and are considered the primary pathway for the emergence of baloxavir resistance (*4*). The PA I38T substitution is the most frequent substitution and has the greatest effect on baloxavir susceptibility (*5*). Influenza A(H1N1)pdm09 (pH1N1) and A(H3N2) viruses with the PA I38T substitution isolated from baloxavir-treated patients show similar replication fitness and pathogenicity to wild-type isolates tested in hamsters and efficiently transmit between ferrets by respiratory droplets (*6*). We have monitored baloxavir susceptibility of seasonal influenza viruses in Japan since the 2017–18 season and reported human-to-human transmission of PA I38T mutant H3N2 viruses in children <10 years of age (*7*,*8*).

Researchers detected a PA I38N substitution in a pH1N1 virus isolated from a patient during a phase 3 clinical trial of baloxavir. That substitution conferred a 24-fold reduction in baloxavir susceptibility in recombinant A/WSN/33(H1N1) and a 10-fold reduction in recombinant A/Victoria/3/75(H3N2) and reduced growth capability in both viruses (*3*,*9*). However, its effect on pH1N1 virus has not been reported. During our 2023–24 surveillance, we detected a PA I38N mutant pH1N1 virus in a 14-year-old patient not treated with baloxavir. Here, we report the in vitro characterization of the PA I38N mutant pH1N1 virus.

## The Study

**Figure 1:**
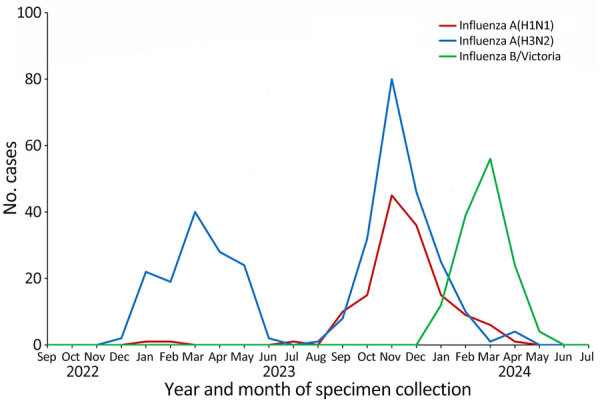
Detection of influenza viruses in Yamagata, Japan, during the 2022–23 and 2023–24 influenza seasons (n = 619). Monthly reports of influenza virus detection by the National Epidemiologic Surveillance of Infectious Diseases (https://www.niid.go.jp/niid/en/survaillance-data-table-english.html) were used for study of influenza A(H1N1)pdm09 virus with reduced susceptibility to baloxavir. Clinical specimens were collected from outpatients who had influenza or influenza-like illness diagnosed.

In March 2024, we detected a pH1N1 virus with the PA I38N substitution (A/Yamagata/103/2024) in Yamagata, Japan. During the 2023–24 season, H3N2 viruses predominated in Japan, followed by pH1N1 and B/Victoria-lineage viruses. In Yamagata, we collected 137 pH1N1, 206 H3N2, and 135 B/Victoria-lineage viruses during the 2023–24 season (Figure 1). 

A 14-year-old patient experienced influenza symptom onset on March 12, 2024, including a high fever and upper respiratory tract infection. A nasal swab specimen was collected on the day of symptom onset. The patient had not received any influenza antiviral drugs before specimen collection. An influenza outbreak occurred at the school attended by the patient, and a class in a different grade was closed on March 13, 2024. No other specimens from the outbreak were available. Ethical approval was not required because this study used data obtained through routine surveillance. 

Deep sequencing analysis performed by using MiSeq (Illumina, https://www.illumina.com) showed that the PA I38N substitution was present at 100% frequency in both the specimen from the patient and the virus isolate. We did not detect any amino acid substitutions associated with reduced inhibition by neuraminidase (NA) inhibitors.

We assessed the susceptibility of the PA I38N mutant and representative pH1N1 viruses, including a PA I38T mutant virus isolated in Yamagata, to baloxavir acid (Funakoshi Co., Ltd., https://www.funakoshi.co.jp) and 4 NA inhibitors approved in Japan: oseltamivir acid (active metabolite of oseltamivir), peramivir, zanamivir, and laninamivir (MedChemExpress, https://www.medchemexpress.com). We determined antiviral drug susceptibilities by using a focus reduction assay (*10*) and a fluorescence-based NA inhibition assay (NA-Fluor Influenza Neuraminidase Assay Kit; Invitrogen, https://www.thermofisher.com/us/en/home/brands/invitrogen.html) and calculated 50% effective concentration (EC_50_) and 50% inhibitory concentration (IC_50_) values by using GraphPad Prism (GraphPad Software, https://www.graphpad.com). To interpret the antiviral drug susceptibility, we applied the criteria proposed by the World Health Organization Expert Working Group on Surveillance of Influenza Antiviral Susceptibility for the Global Influenza Surveillance and Response System (*11**,**12*) by using EC_50_ and IC_50_ fold-change values compared with the median values of pH1N1 viruses isolated during the 2023–24 season in Japan. The criteria for NA inhibitor susceptibility define inhibition of NA activity of influenza A virus as normal (<10-fold increase), reduced (10- to 100-fold increase), or highly reduced (>100-fold increase) (*11*). For baloxavir susceptibility, the provisional criteria define influenza virus susceptibility as normal (≤3-fold increase) or reduced (>3-fold increase) (*12*). The PA I38N and PA I38T mutant viruses showed normal inhibition by all 4 NA inhibitors, but PA I38N exhibited 90-fold higher EC_50_ values and PA I38T exhibited 216-fold higher EC_50_ values to baloxavir compared with the median EC_50_ value of 2023–24 seasonal pH1N1 viruses isolated in Japan (Table 1). Those results indicate that the PA I38N and PA I38T substitutions reduce the susceptibility of pH1N1 virus to baloxavir in vitro.

**Table 1:** Antiviral drug susceptibilities of influenza A(H1N1)pdm09 viruses, Japan, 2024*

Influenza virus	GISAID isolate ID	PA substitution	Specimen collection	Baloxavir† (fold-change)	NA inhibitors‡ (fold-change)			
					Oseltamivir	Peramivir	Zanamivir	Laninamivir
A/Yamagata/103/2024	19183931	I38N	2024 Mar 12	388.20 (90)	0.21 (0.9)	0.11 (1.4)	0.43 (1.1)	0.52 (0.9)
A/Yamagata/333/2023	19045749	I38T	2023 Dec 15	929.18 (216)	0.26 (1.1)	0.08 (1.0)	0.32 (0.8)	0.44 (0.8)
A/Yamagata/122/2023	18744526	None	2023 Sep 21	4.05 (0.9)	0.23 (1.0)	0.08 (1.0)	0.46 (1.2)	0.70 (1.2)
A/Yamagata/127/2023	18744528	None	2023 Oct 2	5.38 (1.3)	0.22 (1.0)	0.08 (1.0)	0.44 (1.2)	0.70 (1.2)
A/Yamagata/135/2023	18799184	None	2023 Oct 15	6.08 (1.4)	0.24 (1.0)	0.08 (1.0)	0.43 (1.1)	0.71 (1.3)
A/Yamagata/177/2023	18853702	None	2023 Nov 4	6.69 (1.6)	0.22 (1.0)	0.06 (0.8)	0.25 (0.7)	0.36 (0.6)
A/Yamagata/292/2023	18987233	None	2023 Dec 4	5.15 (1.2)	0.22 (1.0)	0.09 (1.1)	0.35 (0.9)	0.52 (0.9)
A/Yamagata/312/2023	18987234	None	2023 Dec 11	5.05 (1.2)	0.22 (1.0)	0.11 (1.4)	0.38 (1.0)	0.49 (0.9)
A/Yamagata/336/2023	19045750	None	2023 Dec 15	6.47 (1.5)	0.23 (1.0)	0.09 (1.1)	0.30 (0.8)	0.42 (0.7)
A/Yamagata/104/2024	19201115	None	2024 Mar 27	4.32 (1.0)	0.28 (1.2)	0.09 (1.1)	0.33 (0.9)	0.50 (0.9)

*Influenza A(H1N1)pdm09 for 2023–24 was 4.30 + 2.38 (n = 214) for baloxavir and 0.23 + 0.06 (n = 208) for oseltamivir, 0.08 + 0.02 (n = 208) for peramivir, 0.38 + 0.12 (n = 208) for zanamivir, and 0.56 + 0.14 (n = 208) for laninamivir. EC_50_, 50% effective concentration; fold-change, fold-change in EC_50_ and IC_50_ values compared with the median values of 2023–24 seasonal pH1N1 viruses isolated in Japan; GISAID, GISAID EpiFlu database (http://www.gisaid.org); IC_50_, 50% inhibitory concentration; NA, neuraminidase; PA, polymerase acidic protein. †Mean EC_50_, nmol/L. Mean EC_50_ values of triplicate reactions in a single run were determined by using a focus reduction assay. ‡Mean IC_50_, nmol/L. Mean IC_50_ values of duplicate reactions in a single run were determined by using a fluorescence-based NA inhibition assay.

**Figure 2:**
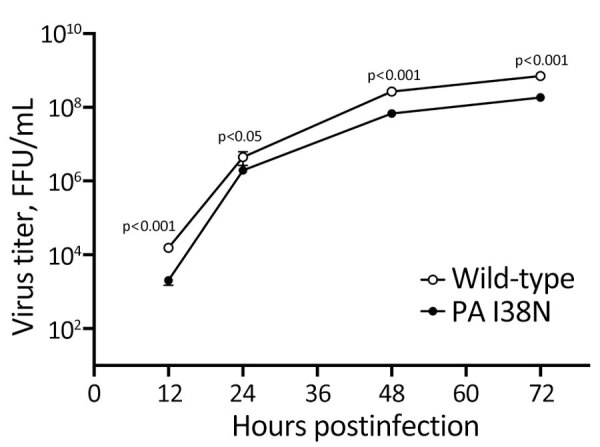
In vitro growth kinetics of the polymerase acidic I38N mutation from study of influenza A(H1N1)pdm09 virus with reduced susceptibility to baloxavir, Japan, 2024. Humanized MDCK cells were infected with the polymerase acidic I38N mutant virus (A/Yamagata/103/2024) or its corresponding wild-type virus (A/Yamagata/336/2023) at a multiplicity of infection of 0.001 focus-forming units per cell. The supernatants were harvested at the indicated times and virus was titrated by using a focus assay. Means (circles) and SDs (error bars) of 4 experiments are shown. p values were calculated by using a *t*-test and fitting a mixed-effects model. FFU, focus-forming units; PA, polymerase acidic.

We then evaluated the effect of the PA I38N substitution on pH1N1 virus growth in vitro (Figure 2) by infecting humanized MDCK cells with the PA I38N mutant or its corresponding wild-type virus at a multiplicity of infection of 0.001 focus-forming units per cell. The wild-type A/Yamagata/336/2023 virus had the most closely related sequences to the PA I38N mutant A/Yamagata/103/2024 virus (Table 2). However, no reports have indicated that those substitutions affect viral replication. The PA I38N mutant virus grew less efficiently than the wild-type virus and showed substantially lower virus titers at all time points. Those results indicated that the PA I38N substitution may negatively affect pH1N1 virus growth in vitro.

**Table 2:** Amino acid differences in influenza A(H1N1)pdm09 viruses with reduced susceptibility to baloxavir, Japan, 2024*

Influenza virus	PA				PB1				PB2				HA			NA			NS1
	38	258	531		617	622	661		152	308	649		6	274		51	383		178
A/Yamagata/103/2024	N	E	K		D	G	T		S	I	I		V	V		E	T		I
A/Yamagata/333/2023	T	E	R		N	G	A		A	V	V		I	M		K	T		V
A/Yamagata/336/2023	I	K	R		D	R	A		A	V	V		V	M		K	I		V

*HA, hemagglutinin; NA, neuraminidase; NS1, nonstructural protein 1; PA, polymerase acidic protein; PB1, polymerase basic protein 1; PB2, polymerase basic protein 2.

## Conclusions

In this study, we showed that the PA I38N mutant pH1N1 virus had reduced susceptibility to baloxavir but remained susceptible to NA inhibitors. Our results indicate that the PA I38N substitution in the pH1N1 virus contributed to a reduction in baloxavir susceptibility, but the reduction in susceptibility was less than that caused by the PA I38T substitution (*3*,*9*).

PA I38 is highly conserved in influenza A and B viruses (*1*). During October 2023–March 2024, medical institutions that serve ≈3.7 million persons in Japan received baloxavir to use for antiviral treatment. The PA I38N substitution may negatively affect the growth capability of the virus in vitro; however, our findings suggest possible transmission of the PA I38N mutant pH1N1 virus from another host harboring the mutant virus, which may have emerged under the selective pressure of baloxavir or as a result of a rare spontaneous mutation.

In Japan, influenza activity was low throughout the COVID-19 pandemic; the first influenza outbreak occurred in the 2022–23 season (*13*). The influenza outbreak in the 2023–24 season was larger than that of 2022–23 (Figure 1). Influenza pH1N1 virus activity peaked in November 2023 and then declined. The PA I38N mutant pH1N1 virus in this study was detected in March 2024. By March, the pH1N1 outbreak was almost over, and no regional spread of the PA I38N mutant pH1N1 virus was observed.

We reported a community cluster of influenza A(H3N2) viruses with reduced susceptibility to baloxavir caused by a PA E199G substitution in Japan in February–March 2023 (*13*). In addition, researchers reported widespread community clusters of pH1N1 viruses with cross-resistance to oseltamivir and peramivir in Australia and Japan (*14*,*15*). Monitoring of antiviral drug susceptibility of influenza viruses is necessary to aid public health planning and clinical recommendations for antiviral drug use.

## References

[1] Omoto S, Speranzini V, Hashimoto T, Noshi T, Yamaguchi H, Kawai M, et al. Characterization of influenza virus variants induced by treatment with the endonuclease inhibitor baloxavir marboxil. Sci Rep. 2018;8:9633. 29941893 10.1038/s41598-018-27890-4 PMC6018108

[2] Uehara T, Hayden FG, Kawaguchi K, Omoto S, Hurt AC, De Jong MD, et al. Treatment-emergent influenza variant viruses with reduced baloxavir susceptibility: impact on clinical and virologic outcomes in uncomplicated influenza. J Infect Dis. 2020;221:346–55. 31309975 10.1093/infdis/jiz244

[3] Ince WL, Smith FB, O’Rear JJ, Thomson M. Treatment-emergent influenza virus polymerase acidic substitutions independent of those at I38 associated with reduced baloxavir susceptibility and virus rebound in trials of baloxavir marboxil. J Infect Dis. 2020;222:957–61. 32253432 10.1093/infdis/jiaa164

[4] Ison MG, Hayden FG, Hay AJ, Gubareva LV, Govorkova EA, Takashita E, et al. Influenza polymerase inhibitor resistance: Assessment of the current state of the art - A report of the isirv Antiviral group. Antiviral Res. 2021;194:105158. 34363859 10.1016/j.antiviral.2021.105158 PMC9012257

[5] Takashita E. Influenza polymerase inhibitors: mechanisms of action and resistance. Cold Spring Harb Perspect Med. 2021;11:a038687. 32122918 10.1101/cshperspect.a038687 PMC8091960

[6] Imai M, Yamashita M, Sakai-Tagawa Y, Iwatsuki-Horimoto K, Kiso M, Murakami J, et al. Influenza A variants with reduced susceptibility to baloxavir isolated from Japanese patients are fit and transmit through respiratory droplets. Nat Microbiol. 2020;5:27–33. 31768027 10.1038/s41564-019-0609-0 PMC13014278

[7] Takashita E, Ichikawa M, Morita H, Ogawa R, Fujisaki S, Shirakura M, et al. Human-to-human transmission of influenza A(H3N2) virus with reduced susceptibility to baloxavir, Japan, February 2019. Emerg Infect Dis. 2019;25:2108–11. 31436527 10.3201/eid2511.190757 PMC6810216

[8] Takashita E, Kawakami C, Ogawa R, Morita H, Fujisaki S, Shirakura M, et al. Influenza A(H3N2) virus exhibiting reduced susceptibility to baloxavir due to a polymerase acidic subunit I38T substitution detected from a hospitalised child without prior baloxavir treatment, Japan, January 2019. Euro Surveill. 2019;24:1900170. 30914078 10.2807/1560-7917.ES.2019.24.12.1900170 PMC6440584

[9] Hashimoto T, Baba K, Inoue K, Okane M, Hata S, Shishido T, et al. Comprehensive assessment of amino acid substitutions in the trimeric RNA polymerase complex of influenza A virus detected in clinical trials of baloxavir marboxil. Influenza Other Respir Viruses. 2021;15:389–95. 33099886 10.1111/irv.12821 PMC8051730

[10] Takashita E, Morita H, Ogawa R, Nakamura K, Fujisaki S, Shirakura M, et al. Susceptibility of influenza viruses to the novel cap-dependent endonuclease inhibitor baloxavir marboxil. Front Microbiol. 2018;9:3026. 10.3389/fmicb.2018.03026 30574137 PMC6291754

[11] World Health Organization. Meetings of the WHO working group on surveillance of influenza antiviral susceptibility – Geneva, November 2011 and June 2012. Wkly Epidemiol Rec. 2012;87:369–74. 23061103

[12] Govorkova EA, Takashita E, Daniels RS, Fujisaki S, Presser LD, Patel MC, et al. Global update on the susceptibilities of human influenza viruses to neuraminidase inhibitors and the cap-dependent endonuclease inhibitor baloxavir, 2018-2020. Antiviral Res. 2022;200:105281. 35292289 10.1016/j.antiviral.2022.105281 PMC9254721

[13] Takashita E, Fujisaki S, Morita H, Nagata S, Miura H, Matsuura Y, et al. A community cluster of influenza A(H3N2) virus infection with reduced susceptibility to baloxavir due to a PA E199G substitution in Japan, February to March 2023. Euro Surveill. 2023;28:2300501. 37768560 10.2807/1560-7917.ES.2023.28.39.2300501 PMC10540515

[14] Hurt AC, Hardie K, Wilson NJ, Deng YM, Osbourn M, Leang SK, et al. Characteristics of a widespread community cluster of H275Y oseltamivir-resistant A(H1N1)pdm09 influenza in Australia. J Infect Dis. 2012;206:148–57. 22561367 10.1093/infdis/jis337 PMC3379839

[15] Takashita E, Kiso M, Fujisaki S, Yokoyama M, Nakamura K, Shirakura M, et al. Characterization of a large cluster of influenza A(H1N1)pdm09 viruses cross-resistant to oseltamivir and peramivir during the 2013-2014 influenza season in Japan. Antimicrob Agents Chemother. 2015;59:2607–17. 25691635 10.1128/AAC.04836-14 PMC4394804

